# Cervical sagittal alignment differences according to scapular rotational posture: A retrospective radiographic study

**DOI:** 10.1097/MD.0000000000050115

**Published:** 2026-08-14

**Authors:** Eun-Mi Jang, Jin-Wook Sung, Byeong-Jin Kim, Hyae-Jeong Byun, Min-Joo Ko, Ui-Jae Hwang, Jae-Seop Oh

**Affiliations:** a Department of Physical Therapy, College of Biomedical Science and Health, INJE University, Gimhae-si, Gyeongsangnam-do, Republic of Korea; b Department of Physical Therapy, Yeonsan Dang Dang Korean Medicine Hospital, Busan, Republic of Korea; c Department of Korean Medicine, Changwon Dang Dang Korean Medicine Hospital, Changwon, Gyeongsangnam-do, Republic of Korea; d School of Disaster and Safety Management, Kyung Hee Cyber University, Seoul, Republic of Korea; e Department of Physical Therapy, College of Health Science, Laboratory of KEMA AI Research (KAIR), Yonsei University, Wonju, Gangwon-do, Republic of Korea.

**Keywords:** cervical sagittal alignment, neck pain, radiographic analysis, scapular rotation

## Abstract

Scapular posture may influence cervical biomechanics because of the anatomical and functional linkage between the cervical spine and the scapulothoracic complex. Although abnormal scapular positioning has been associated with neck pain and altered muscle activity, radiographic evidence describing the relationship between scapular rotational posture and cervical sagittal alignment remains limited. Therefore, this study aimed to compare cervical sagittal alignment parameters between patients with upward rotation (UR) and downward rotation (DR) scapular postures. We retrospectively analyzed cervical spine X-ray images obtained from patients presenting with neck-related symptoms. Participants were classified into UR and DR groups according to scapular rotational posture. Cervical sagittal alignment parameters, including the C0–C2 angle, C2–C7 Cobb angle, C2–C7 sagittal vertical axis (SVA), K-line tilt, and functional spinal unit angles, were measured. Data were analyzed using independent *t*-tests, Pearson correlation analysis, multivariable logistic regression analysis and area under the curve as appropriate. Thirty-six patients (UR: n = 24; DR: n = 12; mean age, 44.64 ± 15.54 years) were included, of whom 58% were female. Compared with the UR group, the DR group showed greater C2–C7 SVA (*P* = .01, *d* = .94), greater K-line tilt (*P* = .03, *d* = .79), and a smaller C4–C5 functional spinal unit angle (*P* = .04, *d* = .74). No significant differences were found in other cervical alignment parameters. Scapular rotational posture was negatively correlated with C2–C7 SVA (*r* = −.562, *P* < .001) and K-line tilt (*r* = −.452, *P* = .007). C2–C7 SVA independently predicted DR posture (odds ratio = 1.109, 95% confidence interval = 1.013–1.214, *P* = .025) and demonstrated acceptable discrimination (area under the curve = 0.75, 95% confidence interval = 0.56–0.94). Downward scapular rotation is associated with altered cervical sagittal alignment, particularly increased anterior cervical translation and segmental changes at the C4–C5 level. These findings highlight a potential biomechanical interaction between scapular rotational posture and cervical sagittal balance, suggesting that evaluation of scapular posture may provide clinically relevant information when assessing cervicoscapular dysfunction in patients with neck pain.

## 1. Introduction

Neck pain is one of the most prevalent musculoskeletal disorders, representing a substantial clinical and socioeconomic burden worldwide, as reported in large population-based and global burden studies.^[1,2]^ Alterations in cervical sagittal alignment have long been implicated in the pathogenesis of neck pain; however, the mechanisms through which postural deviations contribute to cervical dysfunction, particularly in clinical populations with cervical spine disorders, remain incompletely understood.^[3,4]^ Scapular orientation, specifically rotational posture, is an important determinant of cervical biomechanics because of the anatomical and functional interdependence among the cervical spine, cervicothoracic junction, and scapulothoracic complex, as demonstrated in asymptomatic individuals and patients with neck pain.^[5,6]^

The cervical spine maintains horizontal gaze and head stability through coordinated interactions between the upper and lower cervical segments.^[7,8]^ Accordingly, variations in cervical sagittal parameters, including the sagittal vertical axis (SVA), Cobb angle, and craniovertebral orientation, have been associated with altered cervical loading patterns, reduced neck muscle endurance, and increased neck pain and disability, reflected by the higher neck disability index scores in patients with neck pain and degenerative cervical conditions.^[9,10]^ Previous studies have investigated neck pain in relation to forward head posture, thoracic kyphosis, or global postural deviations. However, relatively few studies have examined the influence of scapular rotational posture, including upward rotation (UR) and downward rotation (DR), on cervical sagittal alignment.^[11,12]^ Helgadottir et al^[5]^ reported altered scapular orientation and shoulder girdle alignment in patients with neck pain, suggesting a relationship between scapular posture and cervical dysfunction. Similarly, Andrade et al^[11]^ demonstrated that correction of scapular position in asymptomatic individuals resulted in immediate improvements in cervical rotation range of motion, indicating a functional interaction between scapular alignment and cervical mobility. However, neither study employed radiographic measurements to quantify cervical sagittal alignment parameters.

The scapula functions as a dynamic base for cervical and shoulder musculature. Studies involving asymptomatic individuals and experimental populations have reported that deviations, such as DR, may increase tension in muscles, including the levator scapulae, upper trapezius, and deep cervical extensors.^[5,13,14]^ These muscular adaptations may subsequently influence cervical curvature and sagittal alignment, as described in clinical populations with cervical dysfunction.^[15,16]^ Additionally, previous clinical studies in patients with neck pain and cervicoscapular dysfunction have reported that scapular DR is associated with altered muscle activation patterns and postural adaptations that may increase mechanical loading at the cervicothoracic junction.^[17,18]^ However, objective radiographic evidence supporting this relationship remains limited.^[5,11]^ Moreover, previous investigations have relied largely on observational assessments or electromyographic analyses, often conducted in clinical or experimental populations, which do not directly reflect the structural alignment of the cervical spine.^[11,16]^

Consequently, a clearer understanding of the relationship between scapular rotational posture and cervical sagittal alignment is clinically important for improving postural assessment and guiding rehabilitation strategies in individuals with neck pain. However, quantitative radiographic evidence regarding the association between scapular rotational posture and cervical sagittal alignment remains limited. Therefore, this study aimed to compare cervical sagittal alignment parameters between patients with upward and downward scapular rotation using radiographic measurements. We hypothesized that patients with DR posture would demonstrate greater anterior cervical translation and altered cervical sagittal alignment compared with those with UR posture.

## 2. Methods

### 2.1. Participants

This retrospective cohort study included patients with neck pain who underwent cervical spine and shoulder radiographic examinations at Y Hospital between January 2023 and January 2025. Inclusion criteria were as follows: adult patients aged ≥ 20 years with neck pain lasting more than 3 months, based on the widely accepted definition of chronic pain proposed by the International Association for the Study of Pain and the World Health Organization (International Classification of Diseases, 11th Revision),^[19,20]^ which defines chronic pain as pain persisting for longer than 3 months, thereby ensuring the inclusion of patients with persistent symptoms rather than acute or subacute conditions; availability of cervical spine and shoulder joint radiographs; and adequate radiographic image quality for measurement purposes. Patients were excluded if they had: a history of cervical spine surgery; pain involving joints other than the neck; a history of trauma, tumor, infection, or inflammatory spondyloarthropathy; congenital spinal abnormalities; or inadequate radiographic images precluding accurate measurement.^[21,22]^

**Figure 1.**
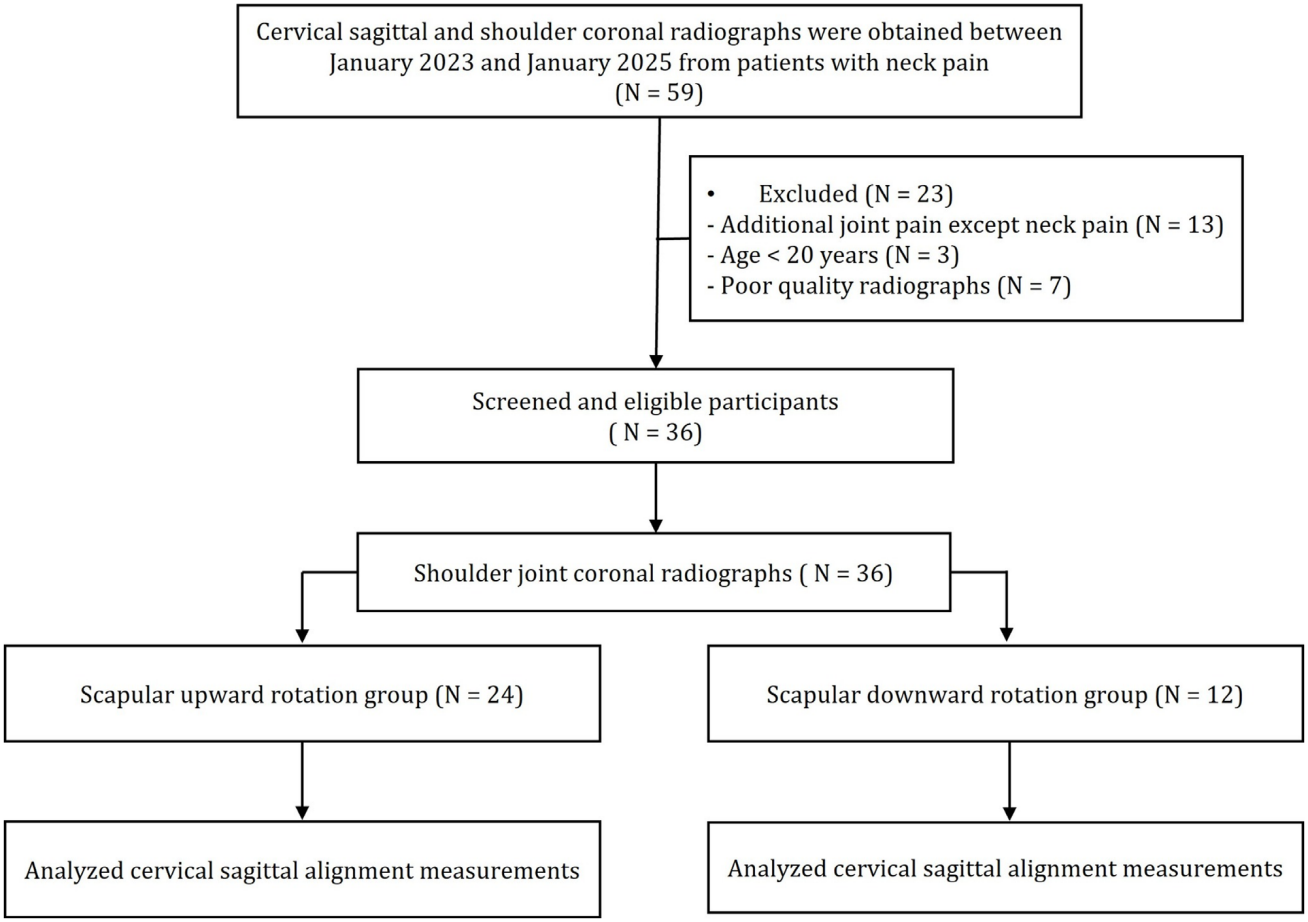
Flowchart of participant recruitment and group classification. Participants were screened according to the inclusion and exclusion criteria and subsequently classified into the UR and DR groups based on scapular rotation measured on radiographs. DR = downward rotation, N = number of patients, UR = upward rotation.

Sample size calculation for an independent 2-sample comparison, with a 2-sided *α* = 0.05 and power = 0.80, indicated that 55 participants would be required to detect a large effect size (Cohen *d* = 0.8) in cervical radiographic parameters. Considering potential exclusions, 59 participants were initially screened. Among them, 13 participants with pain in regions other than the neck, 3 participants under 20 years of age, and 7 participants with poor-quality radiographs unsuitable for analysis were excluded. Consequently, radiographic data from 36 participants were included in the final analysis (Fig. 1). The need for informed consent was waived due to the retrospective nature of the study, which utilized existing medical records and imaging studies. The study protocol was reviewed and approved by the Institutional Review Board of Inje University (Institutional Review Board No. 2025-08-028). This study was reported in accordance with the Strengthening the Reporting of Observational Studies in Epidemiology guidelines.

### 2.2. Radiographic protocol and group classification

All radiographic examinations were performed using standardized positioning protocols with X-ray equipment (SIG-40-525; Ecoray Inc.). Cervical spine and shoulder joint radiographs were obtained with patients in a standing neutral position against a standing post to ensure consistent postural alignment. Cervical spine images were acquired in the sagittal plane (lateral view), while shoulder joint images were obtained in the coronal plane (anteroposterior view). All radiographic examinations were performed by the same licensed radiologic technologist, ensuring consistency in image acquisition across all participants.

**Figure 2.**
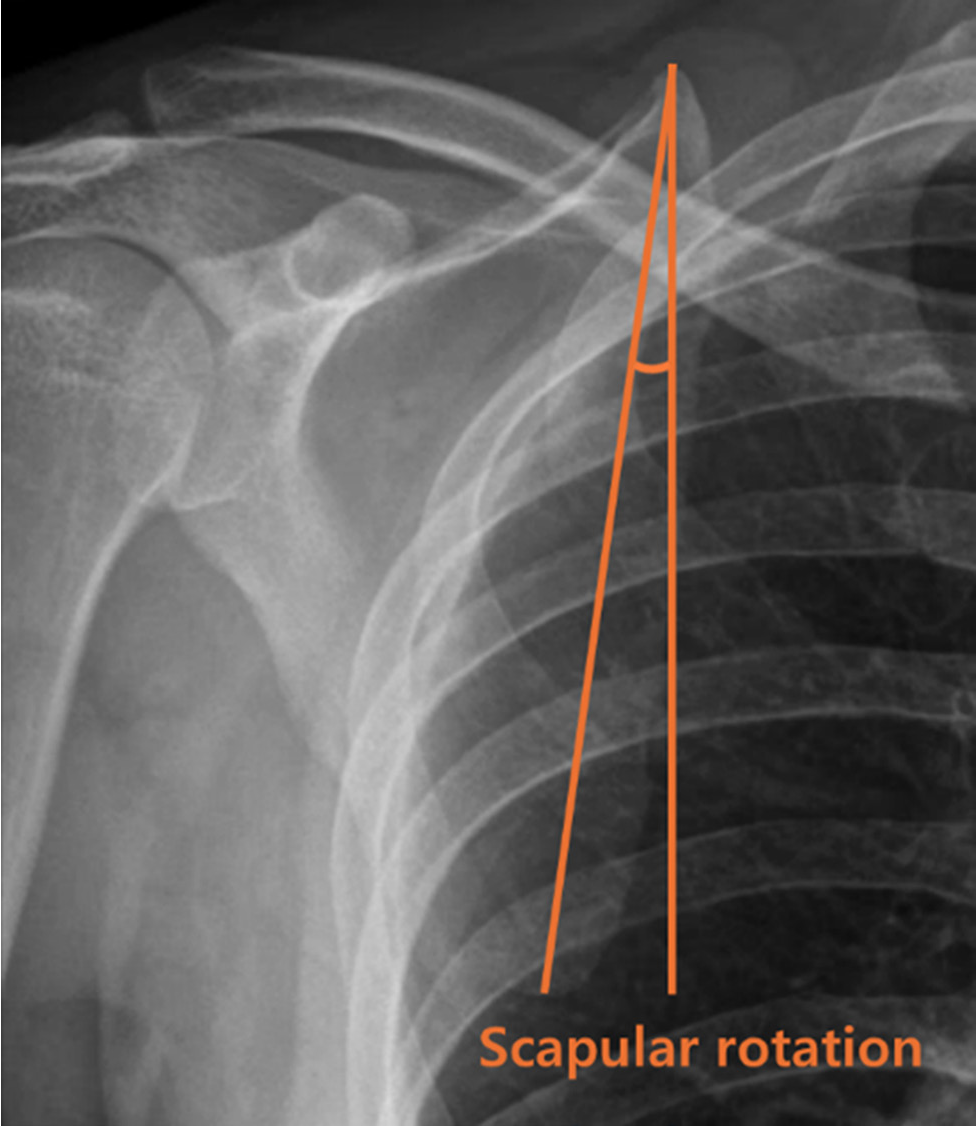
Measurement of scapular rotation angle on an anteroposterior chest radiograph. Scapular rotation angle was determined by measuring the angle formed between a vertical reference line and a line connecting the superior and inferior angles of the scapula.

Scapular rotational position was determined based on the angle between a vertical reference line and a line connecting the superior and inferior angles of the scapula on shoulder anteroposterior radiographs, as described previously (Fig. 2).^[23]^ This method has demonstrated excellent interobserver reliability (intraclass correlation coefficient [ICC] = 0.91) in true anteroposterior views.^[23,24]^ Zero degrees served as the anatomically defined neutral reference of this validated method rather than a statistically derived cut point; positive and negative angles denote upward and DR relative to the vertical, respectively. Participants were classified into 2 groups according to the direction of the measured scapular rotation angle; those with positive values were classified into the UR group and those with negative values into the DR group.^[23]^ The continuous rotation angle was additionally analyzed in the correlation and regression models. The average rotation angle of both scapulae was calculated for each patient to determine group assignment (Fig. 2).

### 2.3. Radiographic measurements

Cervical spine alignment was evaluated using established radiographic parameters measured on lateral cervical radiographs. The measurement methods and corresponding ICC values for each parameter are presented below and were derived from previously published studies rather than independently established in the present study.

**Figure 3.**
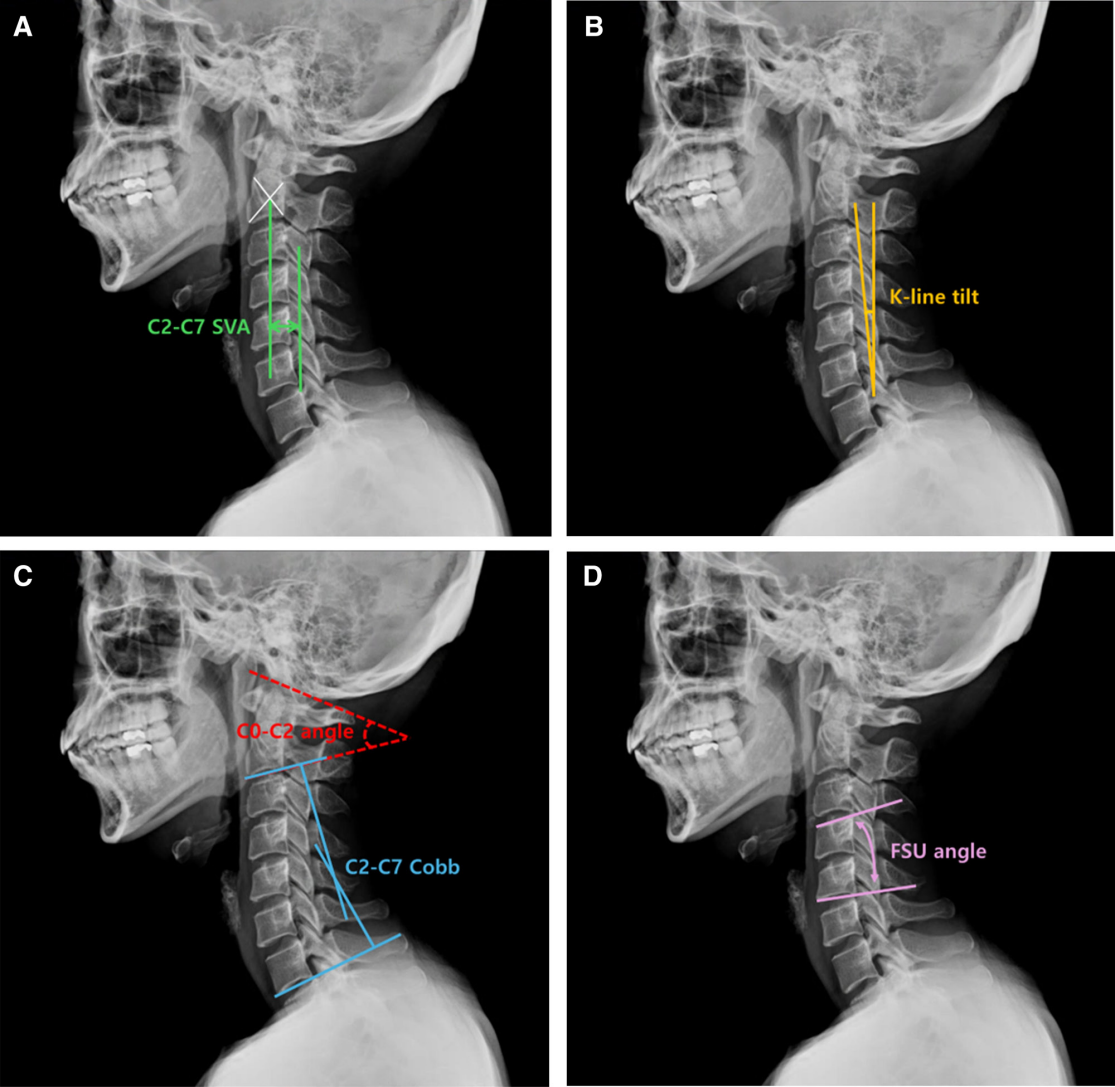
Radiographic parameters used to assess cervical sagittal alignment. (A) C2–C7 SVA, (B) K-line tilt, (C) C0–C2 angle (red line) and C2–C7 Cobb angle (blue line), (D) FSU angle. FSU = functional spinal unit, SVA = sagittal vertical axis.

The C2–C7 SVA was defined as the horizontal distance between a vertical line drawn through the center of the C2 vertebral body and the posterosuperior corner of the C7 vertebral body, as previously described (Fig. 3A).^[25]^ This parameter has demonstrated excellent reliability, with intraobserver ICC values ranging from 0.98 to 0.994 and interobserver ICC values from 0.969 to 0.990.^[26,27]^ K-line tilt was defined as the angle between the K-line, connecting the centers of the spinal canal at C2 and C7, and a vertical reference line (Fig. 3B).^[28]^ Measurement of K-line tilt has demonstrated excellent reliability, with an intraobserver ICC of 0.976 and an interobserver ICC of 0.945.^[27,28]^

The C0–C2 angle was measured between the cranial base line and the inferior endplate of the C2 vertebral body (Fig. 3C, red line).^[29]^ This parameter has shown excellent reliability, with intraobserver ICC values ranging from 0.943 to 0.975 and interobserver ICC values from 0.933 to 0.968.^[27,29]^ The C2–C7 Cobb angle was measured between the inferior endplates of the C2 and C7 vertebral bodies using a standard radiographic method (Fig. 3C, blue line).^[30,31]^ This measurement has demonstrated excellent reliability, with both intraobserver and interobserver ICC values of approximately 0.98.^[30,31]^ Functional spinal unit (FSU) angles were defined as the angles formed between the superior endplate of the upper vertebra and the inferior endplate of the lower vertebra at each intervertebral level from C2–C3 to C6–C7 (Fig. 3D).^[32,33]^ Measurement of FSU angles using the Cobb method has demonstrated good reliability, with an intraobserver ICC of 0.889 and interobserver ICC of 0.874.^[33]^

Interobserver reliability was assessed on a randomly selected subset of 15 images from the present dataset. A second trained observer independently measured the scapular rotation angle, C2–C7 SVA, K-line tilt, and the C4–C5 FSU angle. ICC (2-way random-effects model, absolute agreement) were calculated to evaluate interobserver reliability. Excellent interobserver reliability was observed for all measured variables. The ICCs were 0.953 (95% confidence interval [CI], 0.867–0.984) for the scapular rotation angle, 0.990 (95% CI, 0.970–0.997) for C2–C7 SVA, 0.978 (95% CI, 0.938–0.993) for K-line tilt, and 0.918 (95% CI, 0.773–0.972) for the C4–C5 FSU angle.

All radiographic measurements were performed using a digital picture archiving and communication system (ViewRex 3.0, Techheim). Patient demographic information, including age and gender, was extracted from electronic medical records. All measurements were performed by trained observers using standardized measurement protocols to ensure consistency and minimize variability.

### 2.4. Statistical analysis

All statistical analyses were performed using SPSS Version 29.0.0 (IBM Corp.). Descriptive statistics were calculated for demographic and radiographic variables, and data normality was verified using the Shapiro–Wilk test. Independent samples *t*-tests were applied to compare radiographic cervical alignment parameters between UR and DR. As a sensitivity analysis, the significant between-group differences identified in the primary analysis were reexamined using nonparametric Mann–Whitney *U* tests. Effect sizes were calculated using Cohen *d*, interpreted according to established thresholds (small = 0.20, medium = 0.50, large = 0.80). post hoc power analyses were conducted for significant variables based on observed effect sizes, using a 2-tailed alpha level of 0.05.

Pearson correlation coefficients (*r*) were computed to evaluate associations between scapular rotation angle and radiographic cervical alignment measures. A significance level of *P* < .05 was applied for all inferential analyses. The strength of correlations was interpreted according to conventional criteria (weak < 0.30, moderate 0.30–0.59, strong ≥ 0.60).^[34]^ A 2-tailed *P* < .05 was considered statistically significant for all inferential analyses.

Multivariable logistic regression analysis was performed to evaluate whether cervical sagittal alignment parameters were independently associated with downward scapular rotation. To avoid overfitting given the limited number of events (12 DR cases), a parsimonious multivariable logistic regression model was constructed with C2–C7 SVA as the primary predictor and age as a single covariate. Model goodness-of-fit was evaluated using the Hosmer–Lemeshow test. Odds ratios with 95% CIs were calculated. The discriminative ability of the logistic regression model was assessed using the area under the receiver operating characteristic (ROC) curve (area under the curve [AUC]). No missing data were present for any of the demographic or radiographic variables among the 36 included participants; therefore, no imputation was required.

## 3. Results

A total of 36 patients were included in the analysis, consisting of 24 patients in the UR group and 12 patients in the DR group. Patients in the UR and DR groups had a mean age of 45.33 ± 15.46 and 43.25 ± 13.70 years, respectively. The sex distribution was comparable between groups (58% female and 42% male in both groups), with no significant between-group differences in demographic characteristics (age: *P* = .71; sex: *P* > .99).

Comparisons of radiographic cervical alignment parameters between the UR and DR groups are presented in Table 1. Participants with DR posture demonstrated significantly greater C2–C7 SVA than those in the UR group (21.92 ± 9.95 mm vs 12.94 ± 9.40 mm, *P* = .01, *d* = .94). K-line tilt was significantly larger in the DR group *d* than in the UR group (8.49 ± 6.47° vs 3.83 ± 5.61°, *P* = .03, = .79). At the segmental level, the FSU angle at C4–C5 was significantly smaller in the DR group than in the UR group (2.71 ± 2.68° vs 4.82 ± 2.93°, *P* = .04, *d* = .74). The 95% CIs for the significant between-group differences did not include 0 (Table 1). post hoc power analyses yielded observed power values of 0.73, 0.58, and 0.53 for C2–C7 SVA, K-line tilt, and the FSU angle at C4–C5, respectively.

**Table 1:** Comparison of radiographic cervical alignment parameters between upward rotation and downward rotation groups.

Variables	UR group (n = 24)	DR group (n = 12)	Mean difference (95% CI)	*t*	*P* value	*d*
C2–C7 SVA (mm)	12.94 ± 9.40	21.92 ± 9.95	−8.98 (−15.87–−2.10)	−2.65	.01	.94
K-line tilt (°)	3.83 ± 5.61	8.49 ± 6.47	−4.66 (−8.90–−0.42)	−2.23	.03	.79
C0–C2 angle (°)	36.35 ± 7.42	38.06 ± 6.62	−1.71 (−6.86–3.44)	−0.67	.51	.24
C2–C7 Cobb angle (°)	8.64 ± 5.70	8.66 ± 4.57	−0.02 (−3.87–3.83)	−0.01	.99	.00
FSU angle C2–C3 (°)	11.84 ± 7.20	10.63 ± 6.46	1.21 (−3.80–6.21)	0.49	.63	−.17
FSU angle C3–C4 (°)	4.42 ± 3.34	3.67 ± 2.44	0.75 (−1.46–2.97)	0.69	.49	−.24
FSU angle C4–C5 (°)	4.82 ± 2.93	2.71 ± 2.68	2.11 (0.06–4.16)	2.09	.04	.74
FSU angle C5–C6 (°)	4.70 ± 4.00	3.03 ± 1.66	1.67 (−0.79–4.13)	1.38	.18	−.49
FSU angle C6–C7 (°)	4.99 ± 3.31	4.61 ± 4.05	0.38 (−2.18–2.95)	0.30	.76	−.11

Values are presented as mean ± SD unless otherwise indicated. Mean differences were calculated as UR − DR.

CI = confidence interval, DR = downward rotation, FSU = functional spinal unit, n = number of patients, SD = standard deviation, SVA = sagittal vertical axis, UR = upward rotation.

To examine continuous associations beyond the dichotomous group comparison, Pearson correlation analyses were performed between the scapular rotation angle and each cervical alignment parameter (Table 2). Significant negative correlations were found between the scapular rotation angle and C2–C7 SVA (*r* = −.562, *P* < .001) and K-line tilt (*r* = −.452, *P* = .007). In contrast, no significant correlations were observed between scapular rotation angle and C0–C2 angle, C2–C7 Cobb angle, and any of the FSU angles from C2–C3 to C6–C7 (all *P* > .05). C2–C7 SVA and K-line tilt were highly correlated with each other (*r* = .961, *P* < .001), indicating substantial overlap between these measures of cervical sagittal alignment. As a sensitivity analysis, the significant between-group differences identified in the primary analysis were reexamined using nonparametric Mann–Whitney *U* tests. Significant differences remained for C2–C7 SVA (*P* = .016) and the C4–C5 FSU angle (*P* = .018), whereas K-line tilt showed a similar directional trend but was no longer statistically significant (*P* = .062).

**Table 2:** Pearson correlation coefficients between scapular rotation angle and cervical alignment variables.

	Scapular rotation angle	C2–C7 SVA	K-line tilt	C0–C2 angle	C2–C7 Cobb angle (°)	FSU angle C2–C3	FSU angle C3–C4	FSU angle C4–C5	FSU angle C5–C6	FSU angle C6–C7
Scapular rotation angle (°)	1									
C2–C7 SVA (mm)	−.562**	1								
K-line tilt (°)	−.452**	.961**	1							
C0–C2 angle (°)	−.246	.546**	.517**	1						
C2–C7 Cobb angle (°)	−.075	−.110	−.086	−.324	1					
FSU angle C2–C3 (°)	.021	.105	.151	−.070	.199	1				
FSU angle C3–C4 (°)	.057	.176	.154	.219	−.162	.121	1			
FSU angle C4–C5 (°)	.207	−.025	−.137	.117	−.228	−.122	.232	1		
FSU angle C5–C6 (°)	.115	−.294	−.264	−.246	.143	−.034	.206	.097	1	
FSU angle C6–C7 (°)	.046	−.229	−.247	−.152	.204	−.241	−.250	.215	.202	1

Pearson correlation coefficients (*r*) were calculated between scapular rotation angle and cervical alignment variables.

FSU = functional spinal unit, SVA = sagittal vertical axis.

** *P* < .01 (2-tailed).

**Figure 4.**
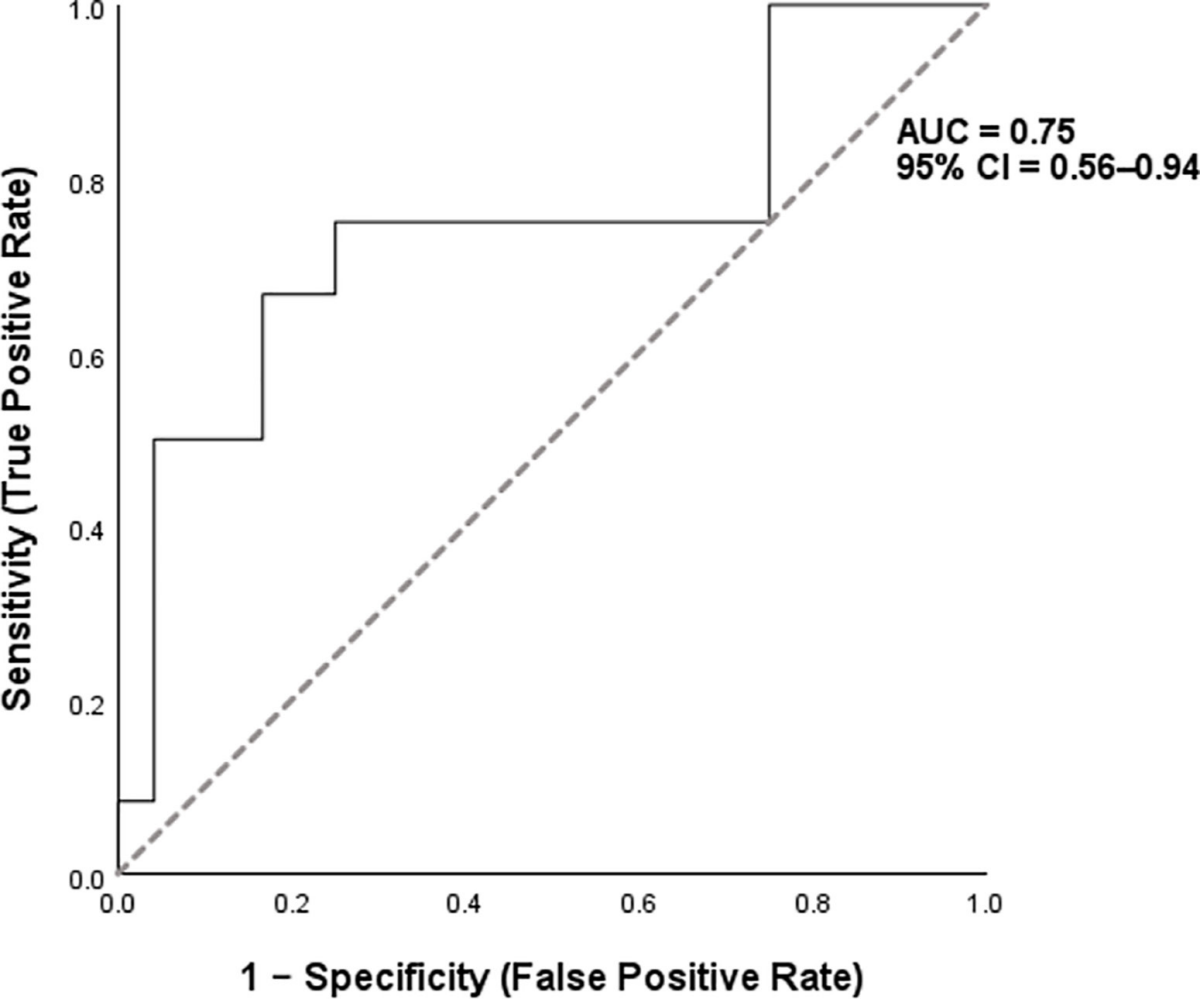
ROC curve analysis of C2–C7 SVA. The ROC curve demonstrates the discriminative ability of C2–C7 SVA for differentiating downward and upward scapular rotation. AUC = area under the curve, CI = confidence interval, ROC = receiver operating characteristic, SVA = sagittal vertical axis.

Multivariable logistic regression analysis was performed to identify predictors of downward scapular rotation (Table 3). In the parsimonious logistic regression model, C2–C7 SVA remained an independent predictor of downward scapular rotation after adjustment for age (odds ratio = 1.109, 95% CI = 1.013–1.214, *P* = .025). This indicates that greater anterior cervical translation was associated with a higher likelihood of downward scapular rotation. Age was not significantly associated with downward scapular rotation (*P* = .930). The model demonstrated adequate goodness-of-fit (Hosmer–Lemeshow *χ*^2^ = 8.610, *df* = 7, *P* = .282). ROC curve analysis of C2–C7 SVA demonstrated acceptable discriminative ability for differentiating individuals with DR from those with UR (AUC = 0.75, 95% CI = 0.56–0.94, *P* = .017) (Fig. 4).

**Table 3:** Multivariable logistic regression analysis for predictors of downward scapular rotation.

Variable	OR	95% CI	*P* value
C2–C7 SVA	1.109	1.013–1.214	.025
Age	0.998	0.951–1.047	.930

To reduce the risk of overfitting given the limited number of downward rotation cases (n = 12), a parsimonious multivariable logistic regression model was constructed including C2–C7 SVA as the primary predictor and age as a covariate. The Hosmer–Lemeshow goodness-of-fit test indicated adequate model fit (*χ*^2^ = 8.610, df = 7, *P* = .282).

CI = confidence interval, n = number of cases, OR = odds ratio, SVA = sagittal vertical axis.

## 4. Discussion

This study identified significant radiographic differences in cervical sagittal alignment between patients demonstrating upward and downward scapular rotational postures. Participants with DR posture showed greater C2–C7 SVA displacement, increased K-line tilt, and a smaller FSU angle at the C4–C5 segment than those with UR posture. No significant differences were observed in the C0–C2, C2–C7 Cobb, or other FSU angles. Pearson correlation analysis also revealed negative associations between scapular rotational posture and C2–C7 SVA and K-line tilt. These findings suggest that clinicians assessing patients with neck pain should routinely evaluate scapular rotational posture as part of their clinical examination, as DR may contribute to altered cervical sagittal loading and therefore represent a modifiable target for rehabilitation.

The association between scapular rotational posture and cervical alignment may be explained by the anatomical and functional continuity of the cervicoscapular muscular system. Muscles such as the levator scapulae, upper trapezius, rhomboids, and deep cervical extensors originate from or attach to the scapula and contribute to maintaining cervical postural equilibrium, as described in anatomical and biomechanical studies.^[35]^ Scapular DR may increase resting tension within these muscles and alter force distribution at the cervicothoracic junction. Such alterations may impair cervical sagittal alignment and extension mechanics, as reported in clinical populations with cervical pain and alignment disorders.^[36,37]^ Thus, our findings may be interpreted in light of previous studies reporting the clinical relevance of scapular DR in patients with neck pain. Increased upper trapezius activity and altered muscle activation patterns have been observed in patients with scapular DR, particularly during low-load tasks, suggesting impaired motor control.^[17]^ In addition, patients with downwardly rotated scapulae have been reported to present with upper cervical pain and headaches and to respond positively to scapular repositioning.^[18]^ Subsequently, increased muscular demand may contribute to a reduction in cervical lordosis and greater reliance on passive stabilizing structures of the cervical spine, as described in patients with cervical alignment abnormalities and sagittal deformities.^[38,39]^

Moreover, the increased SVA displacement observed in the DR group may represent compensatory postural adjustments required to maintain horizontal gaze in the presence of altered scapulothoracic mechanics, as discussed in studies involving spinal alignment and shoulder biomechanics in clinical and theoretical populations.^[40,41]^ Additionally, studies involving both asymptomatic individuals and patients with postural dysfunction have reported that DR may reduce the mechanical efficiency of scapular upward rotators, particularly the serratus anterior and lower trapezius, which may compromise scapulothoracic stability and increase anterior head translation.^[42,43]^ Accordingly, studies comparing symptomatic and asymptomatic individuals have reported that this compensatory positioning may increase external flexion moments acting on the cervical spine and lead to greater activation of cervical extensor muscles and posterior ligamentous structures.^[44]^ In contrast, we hypothesize that UR posture may provide a more stable scapulothoracic platform that could facilitate more balanced cervical loading and potentially reduce compensatory muscular activation; this proposition requires direct testing in future studies. The very high correlation between C2–C7 SVA and K-line tilt (*r* = .961) suggests that these parameters reflect closely related aspects of cervical sagittal alignment. This substantial overlap may explain why K-line tilt was not independently associated with downward scapular rotation when C2–C7 SVA was included in the regression model. Therefore, C2–C7 SVA was retained as the primary predictor in the final parsimonious model.

A significant difference was also observed at the segmental level of the FSU angle at C4–C5. Segmental cervical alignment parameters reflect localized biomechanical adaptations within individual cervical motion segments and may provide additional information beyond global sagittal alignment measures.^[26,28]^ Therefore, the altered FSU angle at C4–C5 observed in the present study may indicate localized mechanical adaptation associated with scapular rotational posture. Given that the C4–C5 segment is located near the cervicothoracic transition zone, structural alterations at this level may reflect biomechanical interactions between cervical alignment and scapulothoracic mechanics. These findings provide additional radiographic evidence supporting the interaction between scapular posture and cervical sagittal alignment.

The multivariable logistic regression analysis further identified C2–C7 SVA as an independent predictor of downward scapular rotation. This finding indicates that greater anterior translation of the cervical spine is associated with a higher likelihood of downward scapular positioning. The present findings extend these observations by suggesting that anterior cervical translation may also be associated with scapular rotational posture through the biomechanical linkage between the cervical spine and the scapulothoracic complex. Because of the cross-sectional design, the direction of this relationship cannot be determined.^[5,13]^ In contrast, K-line tilt and the C4–C5 FSU angle were not retained as independent predictors of scapular rotational posture in the parsimonious model after adjustment for age. These findings suggest that global cervical sagittal translation may play a more important role in cervicoscapular interaction than localized segmental alignment changes. Similar findings have been reported in previous studies showing that global sagittal alignment parameters are more strongly associated with postural adaptations than isolated segmental angles.^[4,16]^ The ROC analysis of C2–C7 SVA demonstrated acceptable discriminative ability (AUC = 0.75, 95% CI = 0.56–0.94),^[45]^ suggesting that cervical sagittal alignment parameters can reasonably distinguish individuals with downward versus upward scapular rotation.

Rehabilitation strategies targeting scapular UR may help reduce mechanical stress on the cervical spine and improve symptoms in individuals with neck pain because scapular mechanics are considered modifiable contributors to cervical sagittal balance. Previous studies in patients with neck pain and degenerative cervical conditions have reported that variations in cervical sagittal parameters, including SVA, Cobb angle, and craniovertebral orientation, are associated with altered cervical loading patterns, reduced neck muscle endurance, and increased neck pain and disability, as reflected by higher neck disability index scores.^[38,42]^ In particular, strengthening of the serratus anterior and lower trapezius muscles has been associated with improved scapular control and optimized scapulothoracic mechanics in individuals with cervicoscapular dysfunction.^[12,15]^ Accordingly, rehabilitation programs incorporating targeted muscle strengthening, postural retraining, and optimization of thoracic alignment may represent effective therapeutic strategies for reducing cervical mechanical stress and improving clinical outcomes in individuals with neck pain.

Several limitations should be considered when interpreting the findings of this study. First, the retrospective single-center study design limits the ability to establish causal relationships between scapular posture and cervical alignment. Second, although age was included as a covariate in the regression analysis, other potential confounding factors such as thoracic posture, occupational habits, and physical activity levels were not fully controlled. Third, the achieved sample size (n = 36; DR, n = 12) fell short of the a priori target of 55 participants required to detect a large effect size (*d* = 0.8) with 80% statistical power. post hoc power analyses yielded observed power values ranging from 0.53 to 0.73, suggesting that smaller effects in secondary outcomes may have gone undetected. In addition, because only 12 participants were classified in the DR group, the stability of the logistic regression model may have been limited. To reduce the risk of overfitting, the final regression model was simplified to include only C2–C7 SVA and age. Nevertheless, the findings of the present study should be interpreted with appropriate caution and confirmed in larger, adequately powered prospective studies. Fourth, the relatively small sample size (n = 36) and the relatively young mean age of participants (44.64 years) may limit the generalizability of the findings to older populations or those with more severe or prolonged neck pain. In addition, neck pain severity was not systematically assessed using validated instruments (e.g., visual analogue scale or numeric rating scale), as this retrospective study relied on existing medical records in which pain intensity scores were not consistently documented. Therefore, future prospective studies should incorporate standardized pain severity assessments to enable a more comprehensive characterization of the study population. Additionally, future studies should include larger prospective multicenter cohorts with longitudinal designs to further clarify the causal relationship between scapular rotational posture and cervical sagittal alignment. The integration of dynamic imaging techniques, 3-dimensional motion analysis, and electromyographic assessments may also provide deeper insight into the complex biomechanical interactions within the cervicoscapular system. Such approaches may ultimately contribute to the development of more targeted rehabilitation strategies for individuals with neck pain associated with scapular postural dysfunction.

## 5. Conclusion

Patients with downward scapular rotation demonstrated distinct radiographic characteristics of cervical sagittal alignment, including greater C2–C7 SVA displacement, increased K-line tilt, and a reduced FSU angle at the C4–C5 segment compared with those with upward scapular rotation. These findings suggest a potential association between scapular rotational posture and alterations in cervical sagittal balance. Assessment of scapular posture may therefore provide clinically relevant information when evaluating cervicoscapular dysfunction in individuals with neck pain. Prospective cohort studies incorporating standardized pain assessment and 3-dimensional motion analysis are warranted to confirm these associations and clarify the directionality of the relationship between scapular rotational posture and cervical alignment.

## Acknowledgments

The authors thank the clinical staff involved in data collection and radiographic acquisition.

## Author contributions

**Conceptualization:** Eun-Mi Jang, Jae-Seop Oh.

**Data curation:** Eun-Mi Jang.

**Formal analysis:** Eun-Mi Jang.

**Funding acquisition:** Jin-Wook Sung.

**Investigation:** Jae-Seop Oh.

**Methodology:** Eun-Mi Jang, Min-Joo Ko.

**Project administration:** Jin-Wook Sung.

**Resources:** Byeong-Jin Kim.

**Software:** Hyae-Jeong Byun.

**Supervision:** Byeong-Jin Kim, Jae-Seop Oh.

**Validation:** Ui-Jae Hwang.

**Visualization:** Eun-Mi Jang.

**Writing – original draft:** Eun-Mi Jang, Ui-Jae Hwang.

**Writing – review & editing:** Ui-Jae Hwang, Jae-Seop Oh.
